# Association between suicidal ideation and suicide: meta-analyses of odds ratios, sensitivity, specificity and positive predictive value

**DOI:** 10.1192/bjo.2018.88

**Published:** 2019-01-31

**Authors:** Catherine M. McHugh, Amy Corderoy, Christopher James Ryan, Ian B. Hickie, Matthew Michael Large

**Affiliations:** Brain and Mind Centre, University of Sydney, Australia; Doctor of Medicine Candidate, School of Medicine, University of Notre Dame Australia, Australia; Clinical Associate Professor, Consultation-Liaison Psychiatrist, Westmead Hospital, Discipline of Psychiatry; and Sydney Health Ethics, University of Sydney, Australia; Co-Director, Health and Policy, The University of Sydney Central Clinical School Brain and Mind Centre Faculty of Medicine and Health, University of Sydney, Australia; Conjoint Professor, School of Psychiatry, University of New South Wales, Australia

**Keywords:** Suicide, mortality, risk assessment

## Abstract

**Background:**

The expression of suicidal ideation is considered to be an important warning sign for suicide. However, the predictive properties of suicidal ideation as a test of later suicide are unclear.

**Aims:**

To assess the strength of the association between suicidal ideation and later suicide measured by odds ratio (OR), sensitivity, specificity and positive predictive value (PPV).

**Method:**

We located English-language studies indexed in PubMed that reported the expression or non-expression of suicidal ideation among people who later died by suicide or did not. A random effects meta-analysis was used to assess the pooled OR, sensitivity, specificity and PPV of suicidal ideation for later suicide among groups of people from psychiatric and non-psychiatric settings.

**Results:**

There was a moderately strong but highly heterogeneous association between suicidal ideation and later suicide (*n* = 71, OR = 3.41, 95% CI 2.59–4.49, 95% prediction interval 0.42–28.1, *I*^2^ = 89.4, *Q*-value = 661, d.f.(*Q*) = 70, *P* ≤0.001). Studies conducted in primary care and other non-psychiatric settings had similar pooled odds to studies of current and former psychiatric patients (OR = 3.86 *v.* OR = 3.23, *P* = 0.7). The pooled sensitivity of suicidal ideation for later suicide was 41% (95% CI 35–48) and the pooled specificity was 86% (95% CI 76–92), with high between-study heterogeneity. Studies of suicidal ideation expressed by current and former psychiatric patients had a significantly higher pooled sensitivity (46% *v.* 22%) and lower pooled specificity (81% *v.* 96%) than studies conducted in non-psychiatric settings. The PPV among non-psychiatric cohorts (0.3%, 95% CI 0.1%–0.5%) was significantly lower (*Q*-value = 35.6, *P* < 0.001) than among psychiatric samples (3.9%, 95% CI 2.2–6.6).

**Conclusions:**

Estimates of the extent of the association between suicidal ideation and later suicide are limited by unexplained between-study heterogeneity. The utility of suicidal ideation as a test for later suicide is limited by a modest sensitivity and low PPV.

**Declaration interest:**

M.M.L. and C.J.R. have provided expert evidence in civil, criminal and coronial matters. I.B.H. has been a Commissioner in Australia's National Mental Health Commission since 2012. He is the Co-Director, Health and Policy at the Brain and Mind Centre (BMC) University of Sydney. The BMC operates an early-intervention youth services at Camperdown under contract to Headspace. I.B.H. has previously led community-based and pharmaceutical industry-supported (Wyeth, Eli Lily, Servier, Pfizer, AstraZeneca) projects focused on the identification and better management of anxiety and depression. He is a Board Member of Psychosis Australia Trust and a member of Veterans Mental Health Clinical Reference group. He was a member of the Medical Advisory Panel for Medibank Private until October 2017. He is the Chief Scientific Advisor to, and an equity shareholder in, InnoWell. InnoWell has been formed by the University of Sydney and PricewaterhouseCoopers to administer the $30 M Australian Government Funded Project Synergy. Project Synergy is a 3-year programme for the transformation of mental health services through the use of innovative technologies.

Health professionals are expected to have the skills to assess and manage patients with suicidal thoughts.^1^ The presence of thoughts of suicide and the expression of suicidal ideation are signs of significant distress, and all patients who express suicidal ideation require a careful clinical assessment^2^^,^^3^ leading to a comprehensive treatment plan.^3^ In addition, suicidal ideation is sometimes considered to be the most important sign of short-term suicide risk,^4^ and it has been argued that questions about suicidal ideation play a crucial role in suicide screening.^5^^,^^6^

Despite the undoubted clinical importance of suicidal ideation, meta-analysis has only been used recently to estimate the statistical strength of suicidal ideation as a test for later suicide. Several meta-analyses have highlighted the modest strength of the association between suicidal ideation and later suicide^7^^–^^10^ and one has quantified the positive predictive value (PPV).^8^ To date, no meta-analysis has examined the sensitivity and specificity of suicidal ideation for suicide. Knowledge of the sensitivity of suicidal ideation for later suicide is particularly important because screening tests should be sufficiently sensitive, such that a high proportion of affected people screen positive.^11^ Moreover, existing meta-analyses of the association between suicide and suicidal ideation have been limited to studies with a cohort design,^9^ only included patients with particular diagnoses^7^ or included studies where suicidal ideation was established post-mortem using psychological autopsy methods.^8^

We report a meta-analysis of cohort and case–control studies that examined the presence or absence of expressed suicidal ideation among those who later did or did not die by suicide.

## Aims and hypotheses

The first aim of this study was to calculate a comprehensive set of measures of prediction of suicidal ideation for later suicide, including of effect size as measured by the pooled odds ratio (OR), sensitivity, specificity, meta-analytically derived receiver operator curve (ROC) and area under the curve (AUC) and the PPV. The second aim was to explore the extent of, and moderators of, between-study heterogeneity in these predictive measures. We hypothesised that suicidal ideation would be a more sensitive and specific test for suicide in non-psychiatric settings, where patients with suicidal ideation can be expected to have fewer additional and potentially confounding risk factors.

## Methods

We conducted a registered meta-analysis (Prospero 42017059236) according to the Preferred Reporting Items for Systematic Reviews and Meta-Analyses (PRISMA) guidelines (see supplementary Table 1 available at https://doi.org/10.1192/bjo.2018.88).^12^

### Searches

**Fig. 1:**
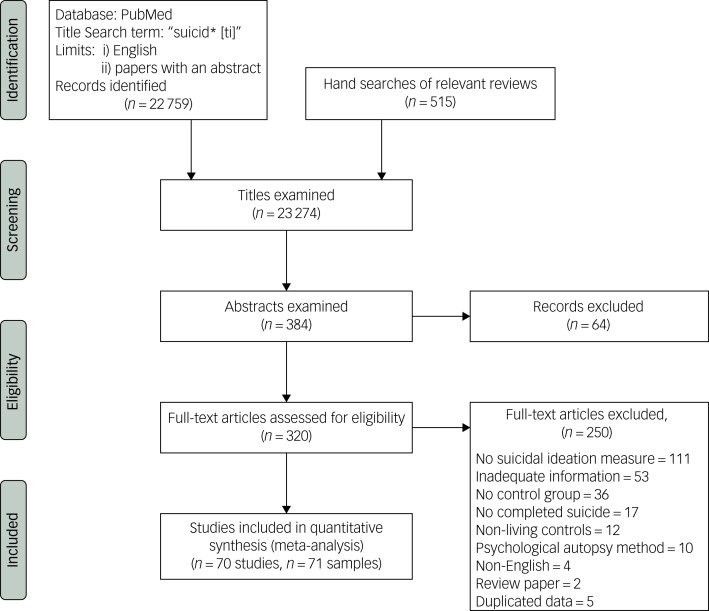
Flow chart of searches.

Multiple search strategies were initially explored using the databases Medline, Embase and PsycINFO from inception to January 2017. These searches yielded an excessive number of titles when the term ‘suicide’ was used as a keyword (>200 000) or in the title (>70 000). Moreover, placing limits in the searches using relevant search terms (such as ‘ideation’ or ‘mortality’) missed numerous relevant papers that were known to the authors through earlier hand searching. However, PubMed indexed all but one English-language publication located by searches of multiple databases in earlier studies.^7^^,^^8^ Therefore, and in order to obtain a large and representative sample of relevant studies, one author (M.M.L.) examined the titles of English-language publications with an accompanying abstract that contained variants of the single term ‘suicide’ (suicid*) in their title and were published in PubMed from inception to 14 September 2017 and hand searched the references list of relevant review articles.^7^^–^^10^^,^^13^^,^^14^ Two authors (M.M.L. and C.M.M.) winnowed the resulting abstracts and full-text publications (Fig. 1).

### Included studies

Studies were included if they reported the expression or non-expression of suicidal ideation among people who later died by suicide or did not, such that all individuals could be classified into the following groups: true positive (those with suicidal ideation and suicide), false positive (those with suicidal ideation but without suicide), false negative (those without suicidal ideation but with suicide) or true negative (those without suicidal ideation or suicide). Studies were included if they provided effect size or other data that was sufficient to enable the calculation of the numbers of people in the true positive, false positive, false negative and true negative categories.

We included studies of patients who had received psychiatric care and people recruited from non-psychiatric settings including primary healthcare, general population samples, military populations and prisons. We excluded studies in which the dependent variable was suicide attempts, or when suicidal ideation was assessed by psychological autopsy methods, when all the controls were deceased or all of the individuals had made suicide attempts. We excluded studies of patients with severe medical illnesses such as malignancies or human immunodeficiency virus.

### Definitions of suicide and suicidal ideation

Two authors (M.M.L. and C.M.M.) independently extracted the numbers of individuals with suicidal ideation among those who had died by suicide and controls and the moderator variables. A third author (A.C.) re-examined the data points. The discrepant points were re-examined by two authors. We accepted the definition of suicidal ideation used in the primary research, acknowledging that the primary research might not have fully reflected differences in the way suicidal ideation was expressed. Studies were coded into four categories according to their definition of suicidal ideation: studies that reported a composite measure of suicidality (inclusive of both suicidal ideation and behaviour); studies that reported suicide plans (including suicide ‘threats’ as reported in the primary studies); studies that recorded a wish to die; and the majority of studies in which the nature of the suicidal ideation or suicidal thoughts was not specified.

### Moderator variables

We collected data about potential moderators of the association between suicidal ideation and suicide; (a) whether the individuals were from psychiatric or non-psychiatric samples; (b) whether the samples consisted of hospital-treated patients; (c) the mean sample age; (d) the duration of follow-up after the assessment for suicidal ideation; (e) whether the study had a cohort or control design; (f) whether patients with suicidal behaviour were regarded as having suicidal ideation; (g) the year of study publication; (h) the proportion of individuals included with suicidal ideation; and (i) the incidence of suicide in the study.

### Assessment of strength of reporting

Two researchers (M.M.L. and C.M.M.) independently assessed the reporting strength (and hence the risk of bias) of each primary study's research using a scale with four further moderator variables (scored 0–4) derived from the Newcastle Ottawa Scale.^15^ One point was allocated according to each of the following criteria: (a) use of a structured method to assess suicidal ideation; (b) collection of data about suicidal ideation in a method that was masked to the patient's suicide (either by a masking method or by electronic recording at the point of assessment); (c) if all the suicides were defined using a mortality database; and (d) inclusion of open verdicts as suicide. Studies counting open verdicts were rated as having stronger reporting because open verdicts are thought to be similar to suicides in some jurisdictions^16^ and because the presence of a history of suicidal ideation might make a coronial verdict of suicide more likely.

### Data synthesis

Random effects meta-analysis was chosen *a priori* for all estimates (OR, sensitivity, specificity, PPV) because of the diversity of study populations and the differences in methods used in the primary research. Between-study heterogeneity in effect sizes was examined using I^2^, *Q*-value statistics and prediction intervals. The possibility of publication bias was assessed using a funnel plot and Egger's regression,^17^ and was quantified using Duval & Tweedie's trim and fill method.^18^ Subgroup analysis (random effects within subgroups) and random-effects meta-regression (method of moments) were used to explore the extent to which between-study heterogeneity was explained by categorical and continuous variables (including the year of publication and the proportion of all participants with suicidal ideation).

Moderator variables (including strength of reporting score items) that were significantly associated with between-study heterogeneity in sensitivity and specificity at *P*≤0.05 were included in multiple meta-regression models. Multiple meta-regression was not used to examine heterogeneity in ORs because of the absence of significant moderator variables. Comprehensive Meta-Analysis (Version 3, Biostat, Englewood NJ) was used for the main analysis and a meta-analytic estimate of the ROC and AUC was calculated using Meta-DiSc.^19^

## Results

### Searches and data extraction

The initial searches yielded 320 potentially relevant papers that were examined in full text. There were 70 papers,^20^^–^^89^ reporting 71 studies in which true positive, false positive, false negative and true negative could be ascertained. Eight differences in the effect size in the independent data extraction were resolved by re-examination of the data (Table 1 and see supplementary Table 2 for the data used in meta-analysis).

**Table 1:** Included studies

Study	Individuals included in the study	Measure of suicidal ideation	Measure of suicide	Odds ratio (95% CI)
Allebeck *et al* (1987)^20^	32 former in-patients with schizophrenia and 64 matched controls	Clinical definition in the medical records	Suicides and open verdicts obtained from a mortality register	2.34 (0.96–5.73)
Al-Sayegh *et al* (2015)^21^	13 suicides among a cohort of 6293 members of the general population	Structured interview for suicidal thoughts and behaviours	Suicides and open verdicts obtained from a mortality register	2.10 (0.65–6.83)
Appleby *et al* (1999)^22^	149 discharged mental health patients and 149 matched controls	Clinical definition in the medical records	Suicides and open verdicts obtained from a mortality register	2.42 (1.52–3.87)
Baader-Matthei *et al* (2004)^23^	32 mental health patients and 32 matched controls	Clinical definition in the medical records	Mortality register	3.82 (1.24–11.80)
Beck *et al* (1999)^24^	30 suicides among a cohort of 3701 mental health out-patients	Scale for suicide ideation	Mortality register	13.84 (5.64–33.98)
Beisser & Blanchette (1961)^25^	71 current mental health in-patients and 71 matched controls	Suicide threats documented in the medical records	Clinically defined	5.61 (2.54–12.41)
Berg (2010)^26^	3 suicides among 20 patients who had received electroconvulsive therapy	Clinical definition in the medical records	Mortality register	1.63 (0.11–22.98)
Bertelsen *et al* (2007)^27^	7 suicides among a cohort of 547 people with first-episode psychosis	Items in the Clinical Assessment in Neuropsychiatry instrument	Mortality register	0.55 (0.07–4.59)
Bickley *et al* (2013)^28^	100 recently discharged mental health patients and 100 matched controls	Retrospectively assessed by treating clinicians	Suicides and open verdicts obtained from a mortality register	2.03 (0.82–5.03)
Blumenthal *et al* (1989)^29^	12 former mental health in-patients and 12 matched controls	Suicide ideation or behaviours assessed with the Present State Examination	Clinically defined	0.50 (0.10–2.60)
Borg & Stahl (1982)^30^	34 first assessed mental health patients and 34 matched controls	Passive suicide ideation determined in a semi-structured interview	Suicides and open verdicts obtained from a mortality register	3.18 (0.76–13.13)
Bradvik & Bergland (1993)^31^	89 melancholically depressed former in-patients and 89 matched controls	Clinical definition in the medical records	Mortality register	1.30 (0.64–2.63)
Cheng *et al* (1990)^32^	55 people with schizophrenia and 52 matched controls	Suicide ideation and behaviours documented in the medical records	Clinically defined	8.52 (1.04–70.07)
Conlon *et al* (2007)^33^	39 mental health patients and 39 matched controls	Suicide ideation documented in the medical records	Identified by a coronial investigator	1.91 (0.51–7.16)
Coryell & Young (2005)^34^	33 suicides among a cohort of 782 patients with a depressive disorder	Schedule for Affective Disorder item 246 > 2	Mortality register	2.84 (0.99–8.19)
Coryell *et al* (2016)^35^	7 suicides among a cohort of 1748 patients with bipolar disorder	Suicidal ideation using the Schedule for Affective Disorder	Clinical with missing individuals assessed with a mortality register	0.63 (0.13–3.11)
Coryell *et al* (2016)^35^	12 suicides among a cohort of 288 patients with bipolar disorder	Suicidal ideation using the Schedule for Affective Disorder	Clinical with missing individuals assessed with a mortality register	2.21 (0.68–7.15)
Crandall *et al* (2006)^36^	449 suicides among a cohort of 218 304 people attending an emergency department	Suicidal ideation documented in the medical records	Mortality register	6.45 (4.81–8.64)
De Hert *et al* (2001)^37^	63 formerly in-patients with schizophrenia and 63 matched controls	Suicidal threats documented in the medical records	Clinically defined	7.92 (3.51–17.89)
Didham *et al* (2006)^38^	221 suicides among primary care patients and 663 matched controls	Suicidal ideation documented in the medical records	Mortality register	17.31 (3.81–78.73)
Dingman & McGlashen (1986)^39^	38 suicides among a cohort of 451 former in-patients	Suicide threat and behaviours documented in the medical records	Clinically defined	2.95 (1.32–6.60)
Dobscha *et al* (2014)^40^	261 veterans treated in primary care and 522 matched controls	Suicide ideation and behaviours documented in the medical records	Mortality register	7.35 (4.45–12.14)
Dong *et al* (2005)^41^	92 in-patient suicides and 92 matched controls	Suicidal ideation documented in the medical records	Suicides and open verdicts obtained from a mortality register	4.68 (2.32–9.44)
Drake *et al* (1986)^42^	15 suicides among a cohort of 104 former in-patients with schizophrenia	Suicidal ideation documented in the medical records	Clinically defined	8.33 (1.77–39.12)
Dutta *et al* (2007)^43^	8 suicides among a cohort of 239 former in-patients with bipolar disorder	Suicidal ideation documented in the medical records	Suicides and open verdicts obtained from a mortality register	2.92 (0.15–58.74)
Farberow *et al* (1966)^44^	218 mental health in-patient suicides and 220 in-patient controls	Suicidal threats documented in the medical records	Clinically defined	7.03 (4.10–12.07)
Fernando & Storm (1984)^45^	22 current or former mental health in-patients and 22 matched controls	Suicidal ideation documented in the medical records	Clinically defined	6.40 (1.65–24.77)
Flood & Seager (1968)^46^	73 former mental health in-patients and 70 matched controls	Suicidal ideation documented in the medical records	Mortality register	0.43 (0.19–0.95)
Fosse *et al* (2017)^47^	36 former mental health in-patients and 120 matched controls	Suicidal ideation item on the Suicide Risk Check List	Mortality register	2.63 (1.20–5.74)
Fowler *et al* (1979)^48^	15 suicides among a cohort of 225 patients with unipolar depression	Suicidal ideation documented in the medical records	Clinically defined	13.41 (0.79–227.6)
Fruehwald *et al* (2004)^49^	220 suicides of prisoners and 440 matched controls	Suicidal threats documented in custodial records	Mortality register	14.86 (8.15–27.08)
Funahashi *et al* (2000)^50^	80 people with schizophrenia and 80 matched controls	Suicidal ideation documented in the medical records	Clinically defined	81.0 (18.45–355.6)
Goldstein *et al* (1991)^51^	46 suicides among a cohort of 1906 former in-patients with affective disorder	Suicidal ideation documented in the medical records	Mortality register	2.96 (1.63–5.36)
Green *et al* (2015)^52^	43 suicides among a cohort of 5200 mental health out-patients	Beck Depression Inventory suicide item >1	Mortality register	5.14 (2.62–10.10)
Hoyer *et al* (2009)^53^	135 current and former mental health in-patients and 135 matched controls	Suicidal ideation documented in the medical records	Mortality register	1.48 (0.91–2.40)
Hunt *et al* (2007)^54^	222 current mental health in-patients and 222 controls	Retrospectively assessed by treating clinicians	Suicides and open verdicts obtained from a mortality register	1.70 (0.94–3.08)
Hunt *et al* (2013)^55^	107 recently admitted mental health patients and 107 controls	Retrospectively assessed by treating clinicians	Suicides and open verdicts obtained from a mortality register	1.56 (0.73–3.35)
Hunt *et al* (2009)^56^	238 recently discharged mental health patients and 238 controls	Retrospectively assessed by treating clinicians	Suicides and open verdicts obtained from a mortality register	2.31 (1.24–4.32)
Hyman *et al* (2012)^57^	24 suicides among a cohort of 4 070 167 military personnel	Systematically collected department of defence data	Mortality register	10.45 (6.92–15.79)
Kan *et al* (2007)^58^	97 recently discharged mental health in-patients and 97 matched controls	Suicidal ideation documented in the medical records	Suicides and open verdicts obtained from a mortality register	1.88 (1.04–3.41)
Kessler *et al* (2015)^59^	68 suicides among a cohort of 316 686 veterans who were former in-patients	Systematically collected department of defence data	Mortality register	2.23 (1.07–4.68)
Khang *et al* (2010)^60^	13 suicides among a cohort of 5414 members of the general population	Suicide ideation item in a national health survey	Mortality register	2.58 (0.87–7.70)
Khanra *et al* (2016)^61^	10 current mental health in-patients and 50 matched controls	Suicidal ideation documented in the medical records	Clinically defined	6.71 (1.12–40.07)
Kim *et al* (2012)^62^	324 depressed veterans and 312 matched controls	Suicide ideation and behaviours documented in the medical records	Mortality register	5.80 (3.86–8.71)
Kjelsberg *et al* (1994)^63^	35 former adolescent mental health in-patients and 35 matched controls	Suicidal ideation documented in the medical records	Mortality register	3.00 (1.03–8.70)
Kleiman & Liu (2014)^64^	25 suicides among a cohort of 19 989 members of the general population	Suicide ideation item in a national health survey	Mortality register	2.82 (0.97–8.22)
Li *et al* (2008)^65^	64 in-patients with schizophrenia and 64 matched controls	Suicidal ideation documented in the medical records	Clinically defined	31.0 (6.98–137.7)
Lin *et al* (2014)^66^	41 current mental health in-patients and 164 controls	Suicidal ideation documented in the medical records	Mortality register	3.44 (1.65–7.17)
Lopez-Morinigo *et al* (2016)^67^	71 people with schizophrenia and 355 controls	Suicide ideation item in a semi-structured risk assessment	Suicides and open verdicts obtained from a mortality register	3.57 (1.41–9.07)
Louzon *et al* (2016)^68^	310 suicides among a cohort of 391 492 veterans	Item 9 of the Patient Health Questionnaire	Mortality register	0.28 (0.22–0.36)
Lukaschek *et al* (2014)^69^	101 former mental health in-patients and 101 matched controls	Suicidal ideas and behaviours documented in the medical records	Railway suicides obtained using a mortality register	1.80 (0.97–3.34)
McEwan *et al* (2010)^70^	3 suicides among a cohort of 138 stalkers	Suicidal ideation documented in the medical records	Clinically defined	4.69 (0.40–55.34)
Mock *et al* (1996)^71^	21 suicides in primary care and 63 matched controls	Suicidal ideation documented in the medical records	Mortality register	3.00 (0.18–50.10)
Motto & Bostrom (1990)^72^	38 suicides among a cohort of 2999 recently admitted mental health patients	Moderate or severe suicidal ideation items from semi-structured interview	Clinically defined	3.96 (1.87–8.39)
Murphy *et al* (1990)^73^	67 patients with alcoholism and 106 controls	Suicidal ideation documented in medical records or survey results	Clinically defined	12.93 (6.15–27.22)
Park *et al* (2017)^74^	96 suicides among a cohort of 8403 recently discharged mental health patients	Suicidal ideation documented in the medical records	Mortality register	2.80 (1.86–4.21)
Powell *et al* (2000)^75^	112 current mental health in-patients and 112 controls	Suicidal ideation documented in the medical records	Suicides and open verdicts obtained from a mortality register	9.94 (5.08–19.45)
Roy (1982)^76^	30 people with schizophrenia and 30 controls	Suicidal ideation documented in the medical records	Clinically defined	3.17 (0.75–13.51)
Salama (1988)^77^	5 suicides among a cohort of 139 current in-patients with schizophrenia	Suicidal ideation documented in the medical records	Clinically defined	23.94 (1.29–422.9)
Sani *et al* (2011)^78^	96 former mental health in-patients and 192 matched controls	Suicidal ideation documented in the medical records	Clinically defined	2.14 (1.24–3.69)
Shah & Ganesvaran (1997)^79^	60 current or recently discharged suicides and 60 matched controls	Suicidal ideation documented in the medical records	Clinically defined	2.31 (1.10–4.85)
Sharma *et al* (1998)^80^	44 current mental health in-patients and 44 matched controls	Suicidal ideation documented in the medical records	Clinically defined	3.40 (1.41–8.19)
Simon *et al* (2013)^81^	46 suicides among a cohort of 84 414 mental health out-patients	Patient Health Questionnaire Item 9	Suicides and open verdicts obtained from a mortality register	7.98 (4.47–14.27)
Sinclair *et al* (2005)^82^	127 current or recently discharged patients with depression and 195 matched controls	Suicidal ideation documented in the medical records	Suicides and open verdicts obtained from a mortality register	1.89 (1.18–3.03)
Sinclair *et al* (2004)^83^	51 current or recently discharged patients with schizophrenia and 82 matched controls	Suicidal ideation documented in the medical records	Suicides and open verdicts obtained from a mortality register	2.88 (1.29–6.47)
Spiessl *et al* (2002)^84^	30 suicides among a cohort of 21 062 mental health in-patients	Suicidal ideation documented in the medical records	Clinically defined	0.98 (0.45–2.14)
Stephens *et al* (1999)^85^	28 suicides among a cohort of 1212 in-patients with schizophrenia	Suicidal ideation documented in the medical records	Clinically defined	5.54 (2.60–11.82)
Thong *et al* (2008)^86^	123 mental health patients and 123 matched controls	Suicidal ideation documented in the medical records	Mortality register	7.99 (3.41–18.70)
Weiser *et al* (2016)^87^	54 suicides among a cohort of 89 049 young men receiving primary mental healthcare	Suicidal ideation and behaviour documented in the medical records	Mortality register	1.64 (0.88–3.06)
Werbeloff *et al* (2016)^88^	8 suicides among a birth cohort of 4914	Suicidal ideation item on a research questionnaire	Mortality register	1.64 (0.20–13.38)
Yim *et al* (2004)^89^	73 recently discharged mental health in-patients and 73 matched controls	Suicidal ideation and behaviour documented in the medical records	Mortality register	2.81 (1.39–5.68)

The earliest study was published in 1961 and the latest in 2017. The median publication date was 2006. In total, 42 studies had a case–control design and 29 were cohort studies. Of the studies, 58 studies were of current or former psychiatric patients including 42 studies of current or former psychiatric in-patients.

In 52 studies suicidal ideation was defined clinically and 19 studies used a structured method. Twelve studies reported a composite measure of suicidality (inclusive of suicide attempts). Four studies reported on suicide plans and two studies recorded a wish to die rather than active suicidal ideation.

The 71 studies included in total of 4 669 303 individuals who had been assessed for suicidal ideation (mean 65 765, median 235) of whom 5 811 died by suicide (mean 81.8, median 49). In total there were 2082 people in the true positive, 125 034 in the false positive, 3729 in the false negative and 4 538 458 in the true negative groups.

### Meta-analyses

#### Meta-analysis of odds ratios

**Fig. 2:**
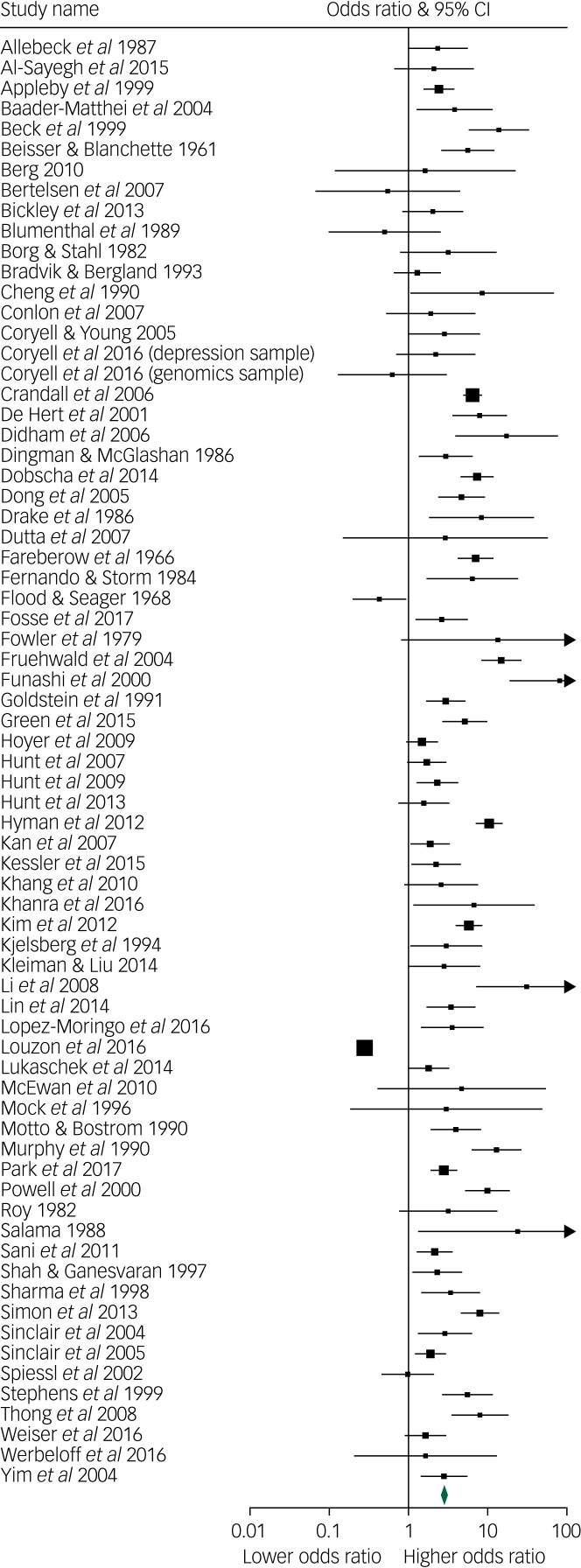
Forest plot of the odds of the association between suicidal ideation and suicide.

The pooled OR of suicide associated with suicidal ideation was 3.41 (95% CI 2.59–4.49; 95% prediction interval (PI) 0.42–28.1). There was very marked between-study heterogeneity, *I*^2^ 89.4, *Q*-value = 661, d.f.(*Q*) = 70, *P*<0.001. The range of ORs was 0.28 to 81, the first quartile,  2.03, median, 2.96 and the third quartile  6.45 (Fig. 2).

**Fig. 3:**
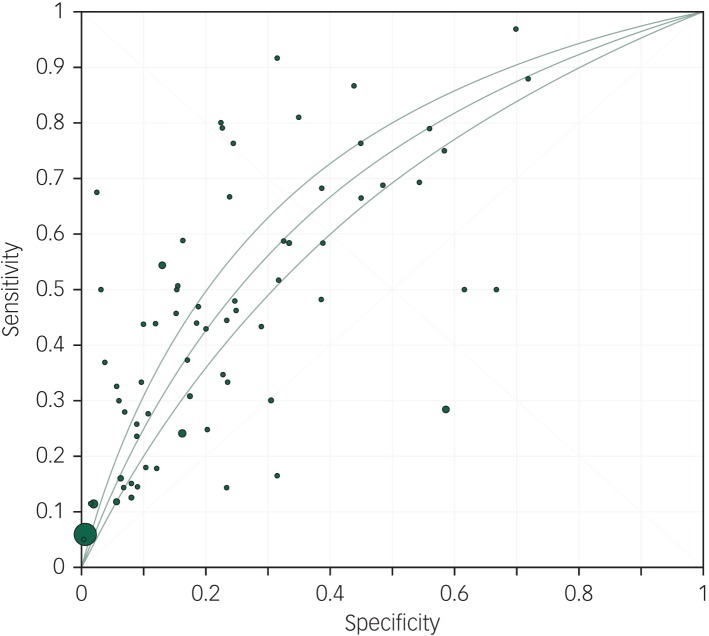
Meta-analytic area under the curve.

**Fig. 4:**
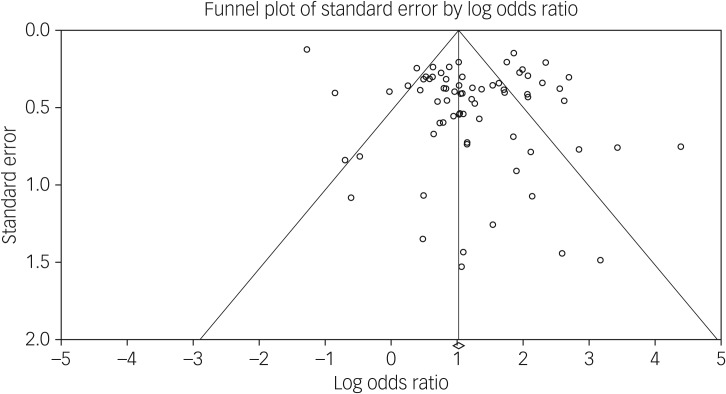
Funnel plot of the odds of the association between suicidal ideation and suicide.

This was associated with a meta-analytic AUC of 0.676 (s.e. = 0.021) (Fig. 3). The funnel plot appeared symmetrical (Fig. 4) but an Egger's regression was borderline significant (intercept 1.55, *t*-value = 2.07, d.f. = 69, *P* = 0.04). Duval & Tweedie's trim and fill did not identify any hypothetically missing studies.

Structured methods to assess suicidal ideation were associated with a non-significantly lower OR (OR = 2.38, 95% CI 1.14–4.99) than clinically defined suicidal ideation (OR = 3.72, 95% CI 2.96–4.67, *Q*-value = 1.28, d.f.(*Q*) = 1, *P* = 0.26). There was no evidence that studies that reported on a composite measure of suicidality spanning suicidal behaviour and suicidal ideation (OR = 3.09, 95% CI 2.02–4.72) differed from studies that simply reported on suicidal ideation (OR = 3.54, 95% CI 2.57–4.88, *Q* = 0.26, d.f.(*Q*) = 1, *P* = 0.61). Suicide plans were more strongly associated with suicide (four studies, OR = 8.51, 95% CI 5.51–13.06) than studies that reported a wish to die (two studies, OR = 3.01, 95% CI 1.49–6.06) and studies that did not specify the degree of intent or planning (65 studies, OR = 3.2, 95% CI 2.41–4.29, *Q*-value = 14.6, d.f.(*Q*) = 2, *P* = 0.001)

Studies that were considered to have stronger reporting on the basis of a total strength of reporting score of ≥2 had a non-significantly weaker association (*Q*-value = 3.58, d.f.(*Q*) = 1, *P* = 0.06) between suicidal ideation and later suicide (*n* = 37, OR = 2.70, 95% CI 1.81–4.01) than studies with a strength of reporting score of <2 (*n* = 34, OR = 4.41, 95% CI 3.20–6.08).

Between-study heterogeneity was not significantly explained by any of our predetermined moderator variables (Table 2). Studies of non-psychiatric samples (*n* = 13, OR = 3.86, 95% CI 2.67–3.97) had similar ORs to studies of psychiatric patients (*n* = 58, OR = 3.23, 95% CI 2.64–3.96; *Q*-value = 0.119, d.f.(*Q*) = 1, *P* = 0.7). The main result and the analyses of moderators were not sensitive to the exclusion of two non-psychiatric studies with very large sample sizes that when combined included over 90% of individuals and 3.5% of the study weight in the random-effects meta-analysis.^36^^,^^57^

**Table 2:** Meta-regression of moderators of the odds ratio of suicidal ideation for later suicide

Variable	Coefficient	Standard error	Lower limit	Upper limit	*Z*-value	*P*
Non-psychiatric samples	0.16	0.37	−0.56	0.87	0.43	0.66
Hospital treated	−0.32	0.29	−0.89	0.25	−1.11	0.26
Mean sample age^a^	−0.41	0.26	−0.92	0.09	−1.62	0.1
Follow-up period^b^	−0.008	0.02	−0.04	0.03	−0.47	0.64
Structured method for suicidal ideation	−0.44	0.29	−1.01	0.14	−1.49	0.14
Use of a mortality database	−0.47	0.30	−1.06	1.22	−1.55	0.12
Cohort study	−0.28	0.29	−0.84	0.28	−0.96	0.34
Included suicide behaviour in the definition	−0.18	0.37	−0.90	0.53	−0.50	0.61
Included open verdicts	−0.18	0.31	−0.80	0.43	−0.59	0.55
Masking of the researchers to suicide	−0.23	0.28	−0.78	0.33	−0.81	0.42
Year of publication	−0.008	0.01	−0.03	0.01	−0.80	0.42
Proportion of individuals with suicidal ideation	−0.94	0.67	−2.25	0.37	−1.40	0.16
Incidence of suicide	0.26	0.51	−0.75	10.27	0.51	0.61

a. 58 studies in the analysis.

b. 68 studies included in the analysis.

#### Meta-analysis of sensitivity

The pooled sensitivity of suicidal ideation for suicide was 41% (95% CI 35–48). There was very high between-study heterogeneity, *I*^2^ = 93.2, *Q*-value = 1033, d.f.(*Q*) = 70, *P*≤0.001. The sensitivity ranged from 0 to 97%, first quartile  25%, median 44% and the third quartile  67%. An Egger's regression was non-significant (intercept 0.60, *t*-value = 0.61, d.f. = 69, *P* = 0.54).

Studies of non-psychiatric populations had a significantly lower sensitivity (*n* = 13, sensitivity  22%, 95% CI 13–53) than studies among psychiatric patients (*n* = 58, sensitivity  46%, 95% CI 40–52; *Q*-value = 10.6, d.f.(*Q*) = 1, *P* = 0.001). This result was not sensitive to the exclusion of the two non-psychiatric studies with very large sample sizes.

Use of a mortality database and more recent publication were associated with a lower sensitivity. Sensitivity was increased among studies of patients in hospital and in studies with a higher proportion of individuals with suicidal ideation (Table 3). The proportion of people with suicidal ideation was the only factor that was independently associated with between-study heterogeneity in a multiple meta-regression (supplementary Table 3). The multiple meta-regression model suggested that the proportion of people with suicidal ideation was the only independent moderator and explained 58% of the between-study heterogeneity in sensitivity.

**Table 3:** Meta-regression of moderators of the sensitivity of suicidal ideation for later suicide

Variable	Coefficient	Standard error	Lower limit	Upper limit	*Z*-value	*P*
Non-psychiatric samples	−1.11	0.31	−1.71	−0.49	−3.55	<0.001
Hospital treated	0.69	0.26	0.19	1.19	2.70	0.007
Mean age^a^	0.14	0.31	−0.48	0.76	0.44	0.66
Follow-up period^b^	−0.02	0.02	−0.02	0.06	0.96	0.34
Structured method for suicidal ideation	−0.09	0.29	−0.67	0.49	−0.30	0.76
Use of a mortality database	−1.01	0.26	−1.51	−0.49	−3.85	<0.001
Cohort study	0.07	0.26	−0.44	0.59	0.28	0.78
Included suicide behaviour in the definition	0.43	0.33	−0.22	1.08	1.29	0.20
Included open verdicts	−0.09	0.29	−0.64	0.48	−0.30	0.77
Masking of the researchers to suicide outcomes	−0.11	0.26	−0.62	0.39	0.44	0.66
Year of publication	−0.02	0.01	−0.04	−0.003	−2.26	0.02
Proportion of individuals with suicidal ideation	5.10	0.50	4.12	6.09	10.1	<0.001
Study incidence of suicide	0.19	0.64	−1.06	1.45	0.3	0.76

a. 58 studies included in the analysis.

b. 68 studies in the analysis.

#### Meta-analysis of specificity

The pooled specificity of suicidal ideation for later suicide was 86% (95% CI 76–92). There was very high between-study heterogeneity, *I*^2^ = 99.9, *Q*-value >10 000, d.f. (*Q*) = 70, *P*≤0.001. The specificity ranged from 28 to 100%, first quartile 69%, median  83% and the third quartile  93%. An Egger's regression was non-significant (intercept  −7.27 *t*-value = 0.74, d.f. = 69, *P* = 0.46).

Studies of non-psychiatric populations had a higher specificity (*n* = 13, specificity 96%, 95% CI 84–99) than studies of psychiatric populations (*n* = 58, specificity  81%, 95% CI 76–85, *Q*-value = 4.78, d.f.(*Q*) = 1, *P* = 0.03). This result was not sensitive to the exclusion of the non-psychiatric studies with very large sample sizes.

Suicidal ideation was a less specific indicator of suicide among studies with a high proportion of people with suicidal ideation and among samples of hospital-treated patients. A multivariate model found that hospital treatment and the proportion of people with suicidal ideation in the study population were significant variables, explaining 79% of the between-study heterogeneity in specificity. (Table 4 and supplementary Table 4).

**Table 4:** Meta-regression tests of potential moderators of the specificity of suicidal ideation for later suicide

Variable	Coefficient	Standard error	Lower limit	Upper limit	*Z*-value	*P*
Non-psychiatric samples	1.65	0.79	0.10	3.20	2.09	0.04
Hospital treated	−1.67	0.62	−2.88	−0.45	−2.69	0.007
Mean sample age^a^	−0.50	0.37	−1.23	0.23	−1.34	0.18
Follow-up period^b^	−0.003	0.03	−0.06	0.06	−0.10	0.92
Structured method for suicidal ideation	0.34	0.53	−0.69	1.39	0.65	0.61
Use of a mortality database	0.37	0.64	−0.89	1.62	0.57	0.57
Cohort study	0.09	0.60	−1.09	1.27	0.15	0.88
Included suicide behaviour in the definition	−0.97	0.87	−2.68	0.74	−1.11	0.26
Included open verdicts	0.05	0.67	−1.25	1.36	−0.08	0.93
Masking of the researchers to suicide outcomes	0.27	0.60	−0.91	1.45	0.46	0.64
Year of publication	0.03	0.02	−0.01	0.08	1.52	0.12
Proportion of individuals with suicidal ideation	−5.73	0.75	−7.21	−4.26	−7.64	<0.001
Study incidence of suicide	−1.05	0.87	−2.80	0.64	−1.23	0.22

a. 58 studies in the analysis.

b. 68 studies included in the analysis.

#### Meta-analysis of PPV

The pooled PPV for suicidal ideation as a test for later suicide among the 29 cohort studies was 1.7% (95% CI 0.9–3.2) over an average duration of follow-up of 9.1 years. There was very high between-study heterogeneity, *I*^2^ = 97.9, *Q*-value = 1186, d.f.(*Q*) = 28, *P*≤0.001. An Egger's regression was non-significant (intercept 2.81, *t*-value = 1.13, d.f. = 27, *P* = 0.27). The PPV among nine non-psychiatric cohorts (0.3% 95% CI 0.1–0.5) was significantly lower (*Q*-value = 35.6, d.f.(*Q*) = 1, *P*<0.001) than among psychiatric samples (3.9% 95% CI 2.2–6.6).

## Discussion

### Main findings

Clinicians sometimes rely on suicidal ideation as a crucial test for short-term suicide risk,^4^ and it has been argued that asking about suicidal ideation could form part of a screening test for later suicide.^5^^,^^6^ Our finding of a pooled OR of suicide associated with suicidal ideation of 3.41 and the meta-analytic AUC of 0.676 indicate a statistical association between suicidal ideation and later suicide with moderate strength.^90^ However, this should be interpreted very cautiously because of the very high and largely unexplained heterogeneity between studies. Moreover, the low PPV of suicidal ideation for suicide, which flows from the low incidence of suicide outcomes, even among psychiatric cohorts, highlights the limitations of suicidal ideation as a practical test of future suicide.

We had hypothesised that suicidal ideation would be a more sensitive and specific test for suicide in non-psychiatric settings, where patients with suicidal ideation might have fewer other risk factors. We found no evidence that suicidal ideation is more strongly associated with suicide in non-psychiatric settings than in psychiatric settings. Contrary to our hypothesis we found that suicide ideation was a less sensitive test for later suicide in non-psychiatric settings, and that the prevalence of suicidal ideation is associated with the sensitivity for later suicide.

Our results can be compared with those of recent meta-analyses of the psychometric properties of suicide risk scales^91^^,^^92^ and multivariate models of suicide risk,^93^ which found similar odds of suicide, sensitivity, specificity and PPV among studies conducted in psychiatric settings as well as similar between-study heterogeneity despite examining quite different study sets. Collectively, these studies highlight a high degree of uncertainty about the statistical strength of commonly used approaches to suicide risk assessment.

The current paper advances knowledge of the association between suicidal ideation and suicide by reporting the pooled sensitivity and specificity of suicidal ideation for suicide. The main finding is the limited sensitivity of suicidal ideation for suicide, such that approximately 60% of people who go on to die by suicide have not expressed suicidal ideation at a specified earlier time. In contrast, the non-expression of suicidal ideation is fairly specific to not dying by suicide in the study period, with only 14% of those not dying by suicide expressing suicidal ideation.

### Implications

Knowledge of the sensitivity and specificity of suicidal ideation for suicide is important because any given OR can be achieved with differing trade-offs between sensitivity and specificity. In this study we found firm evidence that such trade-offs differ between studies, in part as a result of the proportion of individuals expressing suicidal ideation. The proportion of people with suicidal ideation was strongly associated with sensitivity and was inversely associated with specificity, such that similar odds of suicide were found in populations with higher rates of suicidal ideation (i.e. psychiatric patients) and lower rates of suicidal ideation (i.e. non-psychiatric populations).

In general, screening tests should be sensitive enough to capture most cases.^11^ Our study suggests that suicidal ideation is not sensitive enough to be very helpful as a stand-alone screening test for suicide in psychiatric or non-psychiatric settings, a limitation that is compounded by the modest specificity. Clinicians should not assume that patients experiencing mental distress without suicidal ideation are not at elevated risk of suicide. It is notable that our subgroup analysis of non-psychiatric settings found that nearly 80% of people who later die by suicide had not expressed suicidal ideation. Conversely suicidal ideation is somewhat specific to suicide, particularly in settings where the prevalence of suicidal ideation is low. In this sense, the presence of suicidal ideation conveys more salient information about later suicide than the absence of suicidal ideation.

The finding that the proportion of individuals with suicidal ideation was strongly associated with sensitivity and inversely associated with specificity requires some consideration. To some extent this result is a logical consequence of the definition of sensitivity as the proportion of all cases detected – hypothetically, an extremely sensitive test that included the slightest degree of suicidal ideation would likely select almost the entire population and would approach a 100% sensitivity. Although variation in the threshold test for suicidal ideation might have contributed to variation in the prevalence of suicidal ideation between studies in non-extreme examples with typical rates of suicidal ideation, a very wide variation in the proportion of people with suicidal ideation can be associated with a wide variation of sensitivities.

In addition to highlighting uncertainty in the strength of the association between suicidal ideation and suicide, this study highlights an important clinical dilemma about the trade-off between sensitivity and specificity. Although detailed questioning about suicidal ideation is often indicated, for example when there is suspicion of a suicide plan, and such questioning might bring about a greater sensitivity, it might be associated with a higher false positive rate than less detailed questioning.

### Limitations

In addition to the main limitation posed by the extent of between-study heterogeneity, this meta-analysis has a number of other limitations. In most studies there was a lack of clarity about how suicidal ideation was determined and defined. However, this limitation might not have had very much impact on our results because we found little evidence that structured methods of assessing suicidal ideation predicted suicide more accurately than clinical methods. Although it is logical that there may be important threshold issues on the spectrum of severity of suicidal ideation – a possibility supported by our finding of an association between the rate of suicidal ideation and the sensitivity of suicidal ideation for later suicide – there were too few studies that reported different points on the spectrum of ideation to properly explore threshold issues. Another limitation is that we lacked any data about how factors such as gender, comorbid conditions and wider cultural factors might affect the association between suicidal ideation and suicide.

Our results should also be interpreted in light of the knowledge that suicidal ideation can fluctuate over short periods of time.^94^ The primary studies we included measured suicidal ideation at the beginning of a follow-up period, often a follow-up of years. In cases of individuals who died by suicide without earlier suicidal ideation it is likely that suicidal ideation emerged closer to the time of the suicide. It remains to be seen if real time or more temporally proximal measures of suicidal ideation will be more strongly associated with later suicide.^95^

In conclusion, enquiring about suicidal ideation will always be a central skill for mental health professionals, not because suicidal ideation is a meaningful forecast of suicide but because patients who express suicidal ideation are making important communications about their inner world and level of psychological distress. However, clinicians should be aware of the statistical limitations of ideation as a screening tool, and not be lured into either a false confidence generated by an absence of ideation, or determinism about the likelihood of suicide if it is present.

## Supplementary material

For supplementary material accompanying this paper visit http://dx.doi.org/10.1192/bjo.2018.88. click here to view supplementary material
