# Association between suicidal ideation and suicide: meta-analyses of odds ratios, sensitivity, specificity and positive predictive value – ERRATUM

**DOI:** 10.1192/bjo.2019.15

**Published:** 2019-03-04

**Authors:** Catherine M. McHugh, Amy Corderoy, Christopher James Ryan, Ian B. Hickie, Matthew Michael Large

**Keywords:** Suicide, mortality, risk assessment, erratum

The publishers regret to announce that the above paper was published with the incorrect article number.

The correct citation details are as follows:

McHugh CM, Corderoy A, Ryan CJ, Hickie IB, Large MM. Association between suicidal ideation and suicide: Meta-analyses of odds ratios, sensitivity, specificity and positive predictive value. *BJPsych Open* 2019; 5(2): e18. doi:10.1192/bjo.2018.88.

This has now been updated in the original article online.

The publisher sincerely apologises for this error.
