# Supplementary material for: Association between suicidal ideation and suicide: meta-analyses of odds ratios, sensitivity, specificity and positive predictive value
Source: BJPsych Open. 2019 Jan 31;5(2):e18. doi: 10.1192/bjo.2018.88 (PMC6401538; doi:10.1192/bjo.2018.88)
Supplement: Supplementary file 1 [file bjosup.zip › S2056472418000881sup003.docx]

| SM 3. Multiple meta regression of moderators associated with the sensitivity of suicidal ideation for later suicide | | | | | | |
| --- | --- | --- | --- | --- | --- | --- |
|  | Coefficient | Standard error | Low limit | Upper limit | Z -value | P-value |
| Non-psychiatric samples | -0.35 | 0.30 | -0.94 | 0.25 | -1.14 | 0.25 |
| Hospital treated | -0.15 | 0.24 | -0.61 | 0.32 | -0.62 | 0.54 |
| Mortality data base | -0.27 | 0.23 | -0.72 | 0.17 | -1.20 | 0.23 |
| Year of publication | 0.00 | 0.01 | -0.02 | 0.02 | 0.01 | 0.99 |
| Proportion of subjects with suicidal ideation | 4.75 | 0.54 | 3.69 | 5.82 | 8.73 | <0.001 |
| Intercept | -1.56 | 15.52 | -31.97 | 28.85 | -0.10 | 0.92 |
| R-square = 58% | | | | | | |
