# Supplementary material for: Association between suicidal ideation and suicide: meta-analyses of odds ratios, sensitivity, specificity and positive predictive value
Source: BJPsych Open. 2019 Jan 31;5(2):e18. doi: 10.1192/bjo.2018.88 (PMC6401538; doi:10.1192/bjo.2018.88)
Supplement: Supplementary file 1 [file bjosup.zip › S2056472418000881sup004.docx]

| SM 4. Multiple Meta-regression of moderators for between study heterogeneity in specificity of suicidal ideation for suicide. | | | | | | |
| --- | --- | --- | --- | --- | --- | --- |
|  | Coefficient | Standard error | Low limit | Upper limit | Z value | P-value |
| Non-psychiatric | 0.12 | 0.46 | -0.77 | 1.02 | 0.27 | 0.79 |
| Hospital treated | -0.89 | 0.36 | -1.60 | -0.19 | -2.47 | 0.01 |
| Proportion of subjects with suicidal ideation | -4.86 | 0.81 | -6.44 | -3.27 | -6.01 | <0.001 |
| Intercept | 3.73 | 0.38 | 2.99 | 4.47 | 9.92 | <0.001 |
| R-square = 79% | | | | | | |
